# Serum Levels of FGF21, β-Klotho, and BDNF in Stable Coronary Artery Disease Patients With Depressive Symptoms: A Cross-Sectional Single-Center Study

**DOI:** 10.3389/fpsyt.2020.587492

**Published:** 2021-01-21

**Authors:** Yeshun Wu, Zijun Chen, Jiahao Duan, Kai Huang, Bin Zhu, Ling Yang, Lu Zheng

**Affiliations:** ^1^Department of Cardiology, The Third Affiliated Hospital of Soochow University, Changzhou, China; ^2^Department of Cardiology, People's Hospital of Quzhou, Quzhou, China; ^3^Department of Critical Care Medicine, The Third Affiliated Hospital of Soochow University, Changzhou, China; ^4^Comprehensive Laboratory, The Third Affiliated Hospital of Soochow University, Changzhou, China

**Keywords:** stable coronary artery disease, depressive symptoms, fibroblast growth factor 21, β-Klotho, brain-derived neurotrophic factor

## Abstract

**Background:** The incidence of depressive symptoms (DS) in patients with stable coronary artery disease (SCAD) is significantly higher than those in healthy population, and that DS are independent risk factors for cardiovascular events. Previous studies have reported that fibroblast growth factor 21 (FGF21), β-klotho, mature brain-derived neurotrophic factor (mBDNF), and BDNF precursor (proBDNF) play important roles in the pathogenesis and treatment of coronary heart disease and depression. With this in mind, the present study aimed to clarify the relationship between FGF21, β-klotho, mBDNF, and proBDNF and SCAD with comorbid depression, in addition to also exploring the underlying mechanisms of these disease processes.

**Methods:** A total of 116 patients with SCAD and 45 healthy controls were recruited. Patients with SCAD were further divided into two subgroups based on the Zung Self-Rating Depression Scale (SDS), which were characterized as those with no DS (NDS) and those with DS. Baseline data were collected, and serum levels of FGF21, β-klotho, mBDNF, and proBDNF were determined.

**Results:** In SCAD patients, Gensini scores—denoting the degree of coronary arteriostenosis—were significantly greater in the DS group than in the NDS group. There was also a positive correlation between the Gensini scores and the SDS scores. Patients in the SCAD group demonstrated a lower serum FGF21. Serum β-klotho, mBDNF, and mBDNF/proBDNF were also significantly lower in the DS group than in the NDS group. Furthermore, β-klotho and mBDNF were negatively correlated with the SDS scores. Additionally, SCAD patients were divided into lower- and higher-level groups using hierarchical cluster analysis, with the results highlighting that patients in the lower mBDNF group had a higher incidence of DS.

**Conclusions:** The depression score was positively correlated with the severity of coronary artery stenosis, and serum FGF21, β-klotho, mBDNF, and proBDNF were closely related to the development of DS in patients with SCAD. These observations suggest FGF21, β-klotho, mBDNF, and proBDNF as potential diagnostic and/or therapeutic targets for SCAD with co-morbid depression.

## Introduction

Depression is a major cause of disability worldwide and has a huge impact on other chronic diseases (1). An epidemic survey demonstrated that approximately 34.6–51% of Chinese patients with coronary heart disease (CHD) also suffered from depression (2). Of these depression cases, major depressive disorder accounted for 3.1–11.2% (2). To put this into context, the incidence of depression in the general population is only 3.2% (3). The Diagnostic and Statistical Manual of Mental Disorders, 5th Edition (DSM-5) is the gold standard for the diagnosis of clinical depression (4). Furthermore, the depressive symptoms (DS) could be assessed by self-reported clinical scale (4). Currently, approximately 20% of patients that underwent coronary angiography following chest pain demonstrated normal or near-normal coronary arteries, and the chest pain that they had experienced could not be explained by other organic diseases. A study by Christoph et al. enrolled 253 patients to evaluate anxiety, depression, hypochondria, and somatoform disorders using well-validated questionnaires. The results suggested that patients with non-cardiac chest pain were more likely to develop psychopathological symptoms than healthy individuals (5). Stable coronary artery disease (SCAD) is the most common type of ischemic heart disease (6). Although the prognosis for people suffering from SCAD is good, quality of life and health conditions decline drastically in patients suffering from SCAD with co-morbid depression (7, 8). Depression is also one of the independent risk factors for adverse cardiovascular events (7–9).

We previously reported that the incidence of moderate/severe DS in patients with SCAD was 18.8% (10). Further analyses demonstrated that elderly patients were much more likely to experience DS (10). It has been proposed that abnormalities in low-density lipoprotein (LDL), high-density lipoprotein (HDL), and creatinine (Cr) could contribute to DS (10). Interestingly, CHD with co-morbid depression is underpinned by a complex multifactorial process that includes inflammation, endothelial dysfunction, platelet activation, and gut microbiota disturbance, which form a complex pathogenic network (11, 12). Due to limited attention in the clinic, DS are often masked by physical illness. They can also manifest as severe somatic symptoms that are inconsistent with disease severity. For these reasons, diagnosis and appropriate intervention are problematic for clinicians. Therefore, it is of great importance to identify novel, effective biomarkers for the diagnosis and treatment of SCAD with comorbid depression.

Recent studies have indicated that fibroblast growth factor 21 (FGF21) plays an important role in the processes of CHD and depression (13–16). The FGF21 protein is a new member of the FGF protein family and is mainly derived from the liver, kidneys, adipocytes, and cardiomyocytes. The FGF21 protein functions both in the endocrine system and as a cytokine, and it can be released into the circulation to exert its biological effects through specific binding to the co-receptor, β-klotho (17–19). The expression of FGF21 is significantly influenced by β-klotho, which exhibits tissue-specific expression in the liver, heart, and nervous system (20, 21). In addition to its role as a cytokine, FGF21 has an important role in regulating lipid metabolism and inflammation, thereby preserving endothelial function and delaying the development of cardiovascular disease (13–15). Furthermore, Liu and coworkers demonstrated a significant negative correlation between the level of FGF21 in cerebrospinal fluid and depression in male subjects, suggesting that FGF21 has beneficial effects on neuroprotection and emotional regulation (16). Collectively, the above studies indicate that FGF21 is involved in the development of CHD and depression.

Brain-derived neurotrophic factor (BDNF) is an important member of the neurotrophic factor family, which regulates neuronal development and plasticity. BDNF is processed by the Golgi complex from the N-terminal glycosylation precursor protein, BDNF precursor (proBDNF), and released into the extracellular environment (22). Mature BDNF (mBDNF) is a neuroprotective factor that has been associated with neuronal survival, plasticity, and differentiation. Diminished expression of neurotrophic factors represented by mBDNF and the associated impairment of neuroplasticity may directly exacerbate depression (22–25). Interestingly, proBDNF exerts biological effects that are distinct from mBDNF. Specifically, proBDNF upregulates p53 expression and initiates apoptosis through its involvement in the p75 neurotrophin receptor (p75NTR)-activated c-Jun amino-terminal kinase (JNK) pathway, thereby interfering with neurotransmitter release and inhibiting axonal outgrowth (26, 27). Furthermore, mBDNF, as a novel pro-angiogenic factor in CHD, has attracted attention as a contributor to the growth of vascular endothelial cells and proliferation of ischemic endothelial cells. Thus, it appears that mBDNF plays an important role in atherosclerosis and ischemic cardiomyopathy, amongst other diseases (28–30).

In this study, we measured the serum levels of FGF21, β-klotho, mBDNF, and proBDNF in SCAD patients with or without DS and compared this to results from 45 healthy controls (HCs). These findings were used to investigate the relationship between these factors and SCAD with co-morbid depression and to explore the underlying developmental mechanisms.

## Materials and Methods

### Patients and Study Design

A cross-sectional single-center study was conducted using patients from the Third Affiliated Hospital of Soochow University. From July 2017 to October 2018, a total of 116 patients with SCAD were recruited. Forty-five HC subjects were also recruited from the medical examination center. The study was approved by the Ethics Committee of the Third Affiliated Hospital of Soochow University (No. 2017015) and was registered in the Chinese Clinical Trial Registry (ChiCTR1900020594). This study incorporated secondary analyses of clinical trial data from our previous study and used the same registration number (10).

Patients with SCAD were enrolled if they conformed to at least one of the following criteria: (1) clinically diagnosed with myocardial infarction (>3 months); (2) demonstrated at least one coronary artery stenosed by >50% by coronary angiography; (3) demonstrated coronary artery stenosis or myocardial infarction after chest pain; or (4) had undergone coronary artery bypass graft or percutaneous coronary intervention (>3 months).

Patients were excluded from the study if they had experienced: (1) a history of depression or other psychiatric disorders, and on anti-depressant or psychotropic medication; (2) acute myocardial infarction during hospitalization (manifested by electrocardiographic changes and/or elevated myocardial enzymes); (3) myocardial infarction or cardiac surgery in the past 3 months; (4) an acute infectious disease in the month prior to enrollment; (5) other severe cardiovascular diseases (e.g., acute pericarditis, myocarditis, end-stage heart failure, and secondary heart disease); (6) diseases seriously affecting life expectancy (e.g., connective tissue disease, cancer, drug abuse, and dementia); (7) pregnancy; (8) recent major stressful life events; or (9) an inability to complete the depression scale assessment or blood sampling.

### Physical and Clinical Examination

Baseline data were obtained by carrying out interviews, accessing medical records, and assessing age, sex, body mass index (BMI), blood pressure, diabetes history, smoking history, and β-blocker and statin use. Fasting venous blood samples were collected and sent to our laboratory. Alanine aminotransferase (ALT), aspartate aminotransferase (AST), serum total bilirubin (STB), HDL, LDL, albumin/globulins (A/G), blood urea nitrogen (BUN), Cr, and hemoglobin (Hb) were measured. White blood cell (WBC) counts, neutrophil percentage (N%), and lymphocyte percentage (L%) were also assessed. All patients underwent echocardiography to obtain left ventricular ejection fraction (LVEF). All tests were performed and reported by the same physician in the hospital. In addition, all patients underwent coronary angiography via the brachial or radial artery, and the results were interpreted by two experienced cardiologists. The degree of luminal stenosis in the left main, left anterior descending, circumflex, and right coronary arteries were recorded, and the Gensini score was calculated to quantitatively evaluate the degree of coronary artery stenosis.

### Assessment of DS

Patients with SCAD were evaluated for DS using the Zung Self-Rating Depression Scale (SDS) during hospitalization. SDS is one of the most widely used self-reported clinical scale and its validity have been established in clinical depression evaluation (31–33). It has good internal consistency and validity, encompassing most DSM-IV criteria for major depression (32). Consisting of 20 items, the SDS is scored on a four-point scale to assess the psychological and physical symptoms of depression. A standard score is obtained by multiplying the total score by 1.25. Patients with SCAD were further categorized into two subgroups based on their standard scores. These subgroups included patients with no DS (NDS) (score ≤ 52) and those with DS (score ≥ 53).

### Enzyme-Linked Immunosorbent Assay (ELISA)

A volume of 10 ml morning fasting venous blood was collected from the cephalic vein and placed in a non-anticoagulated biochemical test tube. Blood samples were centrifuged at 3,000 r/min for 4 min to obtain serum. A volume of 110 μl of serum was added to each tube and stored at −80°C until measurements were carried out.

Serum levels of FGF21 (Camilo, H-KMLJ31425, the detection range, recovery rate, intra and inter-assay coefficients of variation were 3.75–2,000 pg/ml, 70–110, ≤ 15, and ≤ 15%, respectively), β-klotho (Camilo, H-KMLJ39385, the detection range, recovery rate, intra and inter-assay coefficients of variation were 1.56–20 ng/ml, 70–110, ≤ 15, and ≤ 15%, respectively), mBDNF (Camilo, H-KMLJ39649, the detection range, recovery rate, intra and inter-assay coefficients of variation were 0.78–50 ng/ml, 70–110, ≤ 15, and ≤ 15%, respectively), and proBDNF (Camilo, H-KMLJ31139, the detection range, recovery rate, intra and inter-assay coefficients of variation were 0.312–30 ng/ml, 70–110, ≤ 15, and ≤ 15%, respectively) were determined by ELISA. The operation steps were carried out according to the manufacturer's instructions: (1) aluminum slats were removed from the foil bag after 20 min at room temperature; (2) standard wells, sample wells, and blank wells were set, and standard wells were loaded with 50 μl of standards; (3) 10 μl of sample and 40 μl *diluent* were added to the sample wells; (4) 50 μl of horseradish peroxidase-labeled detection antibody was added to the standard wells and the sample wells; (5) the plate was sealed and incubated at 37°C in a water bath or thermostat for 60 min; (6) the liquid was discarded and washing solution was added to each well before leaving to stand for 1 min; (7) washing solution was removed; (8) steps (6) and (7) were repeated five times; (9) 50 μl of each of substrates A and B were added to each well and incubated at 37°C for 15 min in the dark; and (10) 50 μl of stop solution was added to each well, and the absorbance was measured immediately at 450 nm. The concentration of each factor was obtained according to the optical density-concentration standard curve.

### Statistical Analysis

Statistical analysis was performed using SPSS 24.0 and GraphPad Prism 7.0. Data were expressed as mean ± standard deviation, and each parameter was tested for normality. If the data were normally distributed, the means of the two groups were compared using a *t*-test. If data were not normally distributed, the means of the two groups were compared using the Mann–Whitney *U*-test. Categorical data were expressed as the rate (%), and a chi-squared test was used. A correlation analysis was performed using the Pearson correlation, Spearman correlation and multiple linear regression analysis. A two-sided *p* < 0.05 was considered statistically significant for all tests.

## Results

### Comparison Between the Clinical Characteristics of HC and SCAD Subjects

There were no significant differences in age, gender, blood pressure, AST, A/G, Cr, and N% between the two groups (*p* > 0.05), while the SCAD group had a greater BMI, a greater ALT and WBC, and lower STB, HDL, LDL, BUN, Hb, and L% levels (*p* < 0.05; Table 1).

**Table 1:** Clinical characteristics of HC and SCAD subjects.

**Parameters**	**HC (*n* = 45)**	**SCAD (*n* = 116)**	***P*-value**
Age (years)	61.2 ± 8.7	63.4 ± 9.0	0.166
Male (%)	32(71.1)	84(72.4)	0.869
SBP (mmHg)	135 ± 19	135 ± 18	0.936
DBP (mmHg)	78 ± 11	79 ± 11	0.433
BMI (kg/m^2^)	23.7 ± 1.7	24.6 ± 3.0	0.022
ALT (U/L)	19.4 ± 7.9	25.1 ± 14.4	0.018
AST (U/L)	22.5 ± 7.0	23.7 ± 7.9	0.344
STB (μm/L)	12.8 ± 4.3	10.2 ± 4.4	0.001
HDL (mmol/L)	1.27 ± 0.29	1.05 ± 0.23	<0.001
LDL (mmol/L)	2.56 ± 0.53	2.04 ± 0.69	<0.001
A/G	1.71 ± 0.22	1.64 ± 0.30	0.054
BUN (mg/dl)	5.79 ± 2.10	4.98 ± 1.86	0.019
Cr (μmmol/L)	79.5 ± 11.3	80.1 ± 44.4	0.901
Hb (g/L)	147.4 ± 13.9	138.6 ± 12.3	<0.001
WBC (× 10^9^/L)	5.54 ± 1.31	6.51 ± 1.51	<0.001
N (%)	59.9 ± 7.7	63.2 ± 8.4	0.026
L (%)	33.2 ± 7.1	27.6 ± 8.7	<0.001

*A/G, albumin/globulins; ALT, alanine aminotransferase; AST, aspartate aminotransferase; BMI, body mass index; BUN, blood urea nitrogen; Cr, creatinine; DBP, diastolic blood pressure; Hb, hemoglobin; HC, healthy controls; HDL, high density lipoprotein; L%, lymphocyte percentage; LDL, low density lipoprotein; N%, neutrophil percentage; SCAD, stable coronary artery disease; SBP, systolic blood pressure; STB, serum total bilirubin; WBC, white blood cell. After tested for normality, the means of ALT, AST, A/G, and Cr of the two groups were compared using the Mann–Whitney U-test, while others were compared using a t-test and a chi-squared test (for categorical data)*.

**Figure 1:**
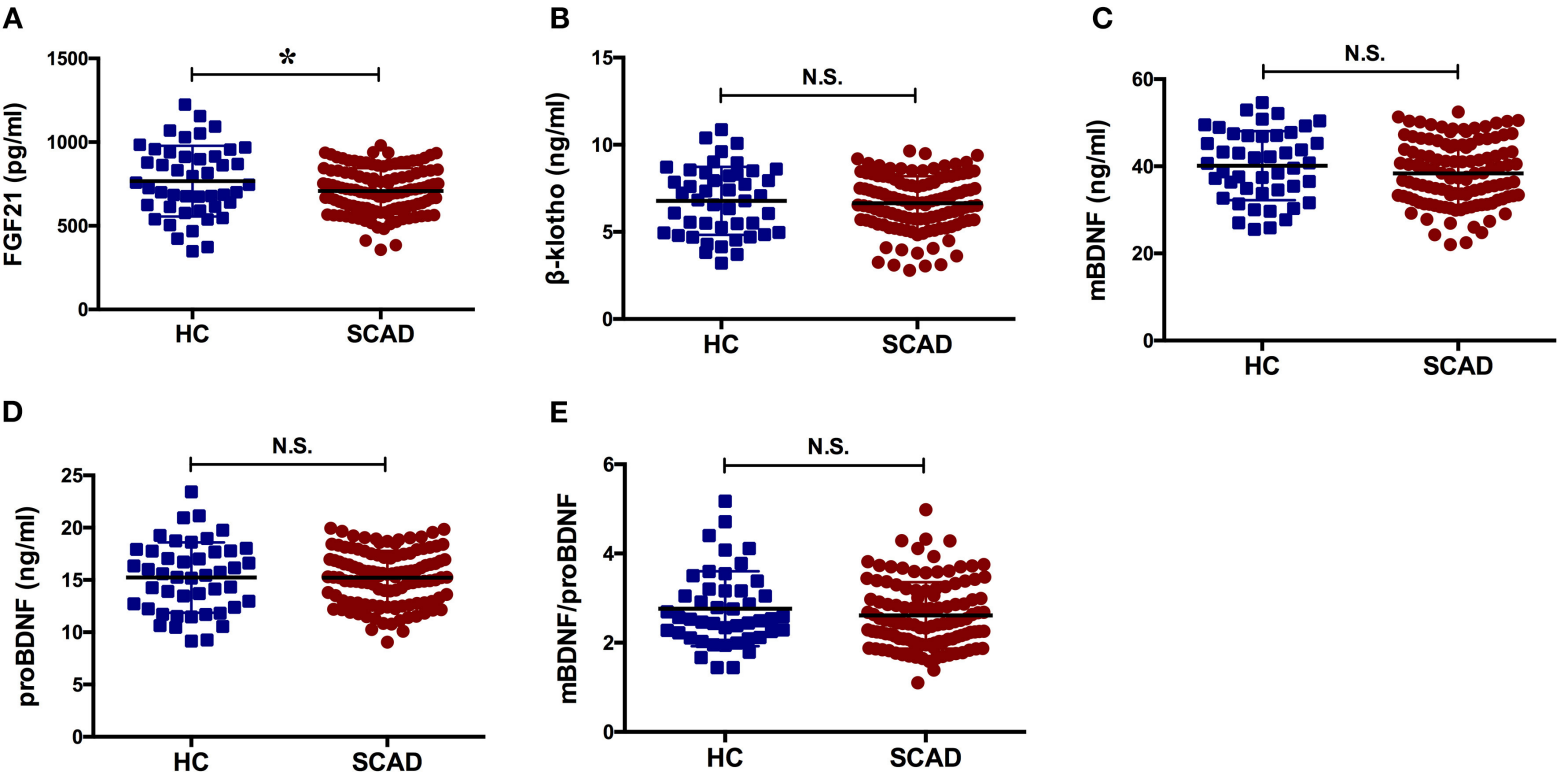
Serum levels of the factors in HC and SCAD. BDNF, brain derived neurotrophic factor; FGF21, fibroblast growth factor 21; HC, healthy controls; N.S., no significance; SCAD, stable coronary artery disease. The means of the two groups were compared using a *t*-test. The concentration of FGF21 in the SCAD group was significantly lower than that observed in the HC group [(707.80 ± 136.40) pg/ml vs. (766.62 ± 211.36) pg/ml, *p* = 0.039].

The concentration of FGF21 in the SCAD group was significantly lower than that observed in the HC group (*p* = 0.039). The serum concentrations of β-klotho, mBDNF, proBDNF, and mBDNF/proBDNF were decreased in the SCAD group. However, no statistically significant difference was identified when compared with the HC group (*p* > 0.05; Figure 1).

### Comparison of the Clinical Characteristics of SCAD Patients With and Without DS

There were no significant differences in age, gender, blood pressure, BMI, diabetes history, smoking history, ALT, AST, STB, HDL, LDL, A/G, BUN, Cr, Hb, L%, N%, LVEF, or β-blocker and statin use between SCAD patients with and without DS (*p* > 0.05). The group with DS had lower WBC counts and greater Gensini scores (*p* < 0.05; Table 2).

**Table 2:** Clinical characteristics of SCAD patients with or without depressive symptoms.

**Parameters**	**Without depressive symptoms (*n* = 30)**	**With depressive symptoms (*n* = 86)**	***P*-value**
Age (years)	62.0 ± 10.5	63.9 ± 8.5	0.382
Male (%)	22 (64.7)	62 (72.1)	0.896
SBP (mmHg)	134 ± 18	135 ± 19	0.868
DBP (mmHg)	80 ± 12	79 ± 10	0.799
BMI (kg/m^2^)	25.0 ± 3.0	24.4 ± 3.0	0.429
Diabetes mellitus (%)	6 (20.0)	32 (37.2)	0.084
Current smoking (%)	10 (33.3)	32 (37.2)	0.704
ALT (U/L)	27.9 ± 17.2	24.1 ± 13.3	0.292
AST (U/L)	25.1 ± 8.7	23.3 ± 7.5	0.287
STB (μm/L)	10.9 ± 3.5	10.0 ± 4.6	0.362
HDL (mmol/L)	1.00 ± 0.21	1.07 ± 0.24	0.120
LDL (mmol/L)	2.12 ± 0.77	2.01 ± 0.66	0.467
A/G	1.72 ± 0.38	1.61 ± 0.25	0.247
BUN (mg/dl)	4.76 ± 1.16	5.06 ± 2.05	0.443
Cr (μmmol/L)	81.4 ± 31.8	79.6 ± 48.2	0.162
Hb (g/L)	138.3 ± 13.3	138.8 ± 12.0	0.859
WBC (× 10^9^/L)	7.03 ± 1.74	6.33 ± 1.39	0.029
N (%)	61.4 ± 6.9	63.8 ± 8.9	0.183
L (%)	29.1 ± 9.5	27.1 ± 8.4	0.284
Medications (%)			
β blockers	7 (23.3)	36 (41.9)	0.070
Statins	13 (43.3)	41 (47.7)	0.681
LVEF (%)	62.1 ± 2.8	61.5 ± 5.4	0.570
Gensini scores	19.72 ± 8.96	30.71 ± 23.40	<0.001

*A/G, albumin/globulins; ALT, alanine aminotransferase; AST, aspartate aminotransferase; BMI, body mass index; BUN, blood urea nitrogen; Cr, creatinine; DBP, diastolic blood pressure; Hb, hemoglobin; HC, healthy controls; HDL, high density lipoprotein; L%, lymphocyte percentage; LDL, low density lipoprotein; LVEF, left ventricular ejection fraction; N%, neutrophil percentage; SCAD, stable coronary artery disease; SBP, systolic blood pressure; STB, serum total bilirubin; WBC, white blood cell. After tested for normality, the means of ALT, AST, A/G, Cr, LVEF, and Gensini scores of the two groups were compared using the Mann–Whitney U-test, while others were compared using a t-test and a chi-squared test (for categorical data)*.

**Figure 2:**
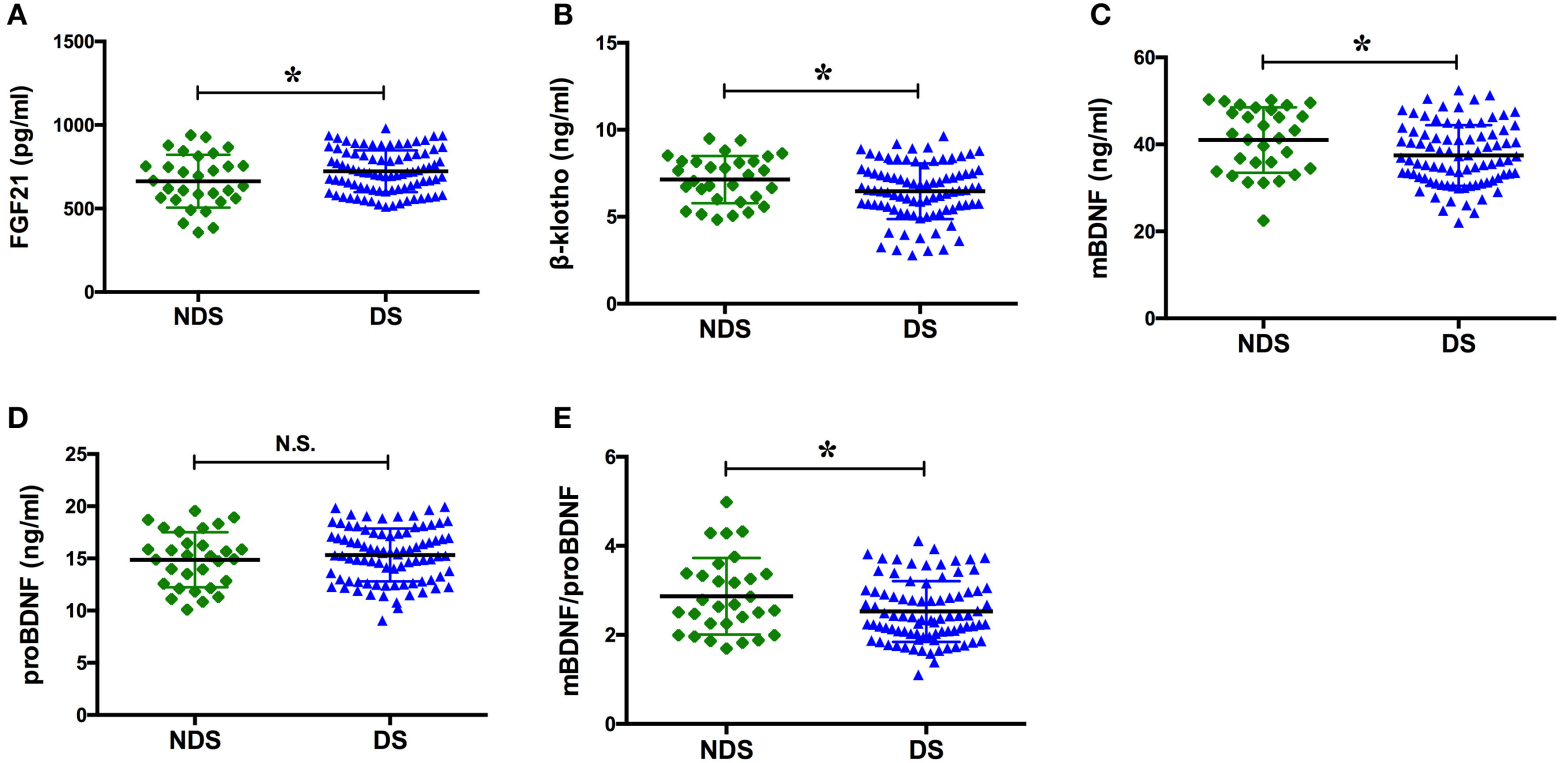
Serum levels of the factors in NDS and DS. BDNF, brain derived neurotrophic factor; DS, depressive symptoms; FGF21, fibroblast growth factor 21; NDS, no depressive symptoms; N.S., no significance. The means of the two groups were compared using a *t*-test. The serum concentration of FGF21 in the group with DS was significantly greater than that observed in the group with NDS [(723.23 ± 125.49) pg/ml vs. (663.58 ± 157.78) pg/ml, *p* = 0.039], while the serum concentrations of β-klotho, mBDNF, and mBDNF/proBDNF were significantly lower [(6.46 ± 1.60) ng/ml vs. (7.14 ± 1.35) ng/ml, *p* = 0.041; (37.47 ± 6.98) ng/ml vs. (41.00 ± 7.50) ng/ml, *p* = 0.021; and (2.53 ± 0.68) vs. (2.86 ± 0.86), *p* = 0.029, respectively]. *p < 0.05.

The serum concentration of FGF21 in the group with DS was significantly greater than that observed in the group with NDS (*p* = 0.039), while the serum concentrations of β-klotho, mBDNF, and mBDNF/proBDNF were significantly lower (*p* = 0.041, *p* = 0.021, and *p* = 0.029, respectively). ProBDNF levels were increased in the group with DS, but there was no statistically significant difference when compared with the group with NDS (*p* > 0.05; Figure 2).

### Correlation Analysis Between Serum Concentrations, Gensini Scores, and the SDS

**Figure 3:**
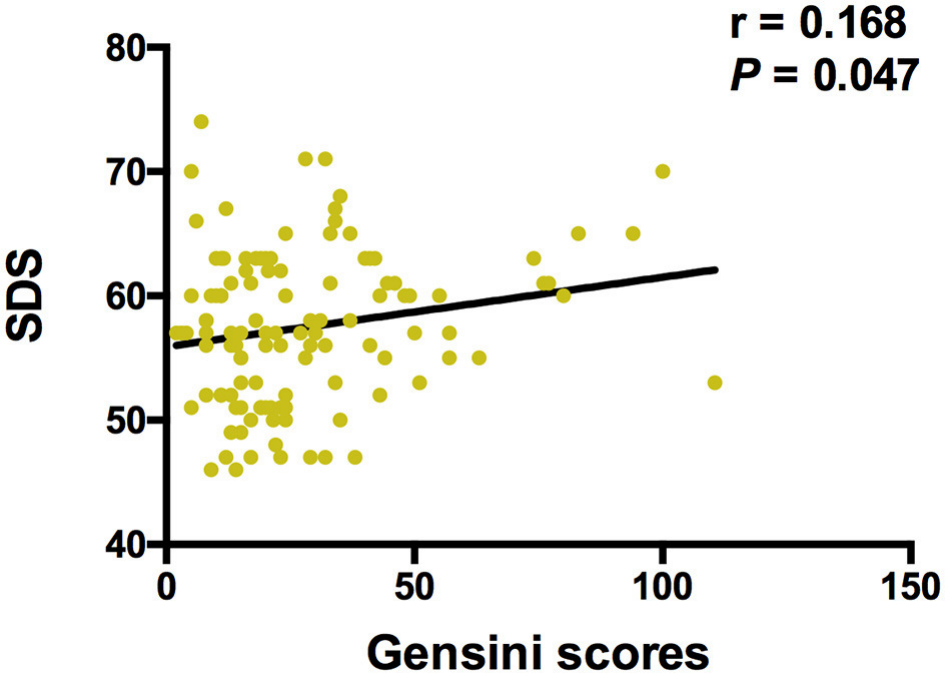
Spearman correlation analysis between Gensini scores and SDS. SDS, self-rating depression scale. The correlation analysis was performed using the Spearman correlation analysis. There is a positive correlation between the Gensini scores and the SDS scores, and the correlation coefficient (r) was 0.168 (*p* = 0.047).

**Figure 4:**
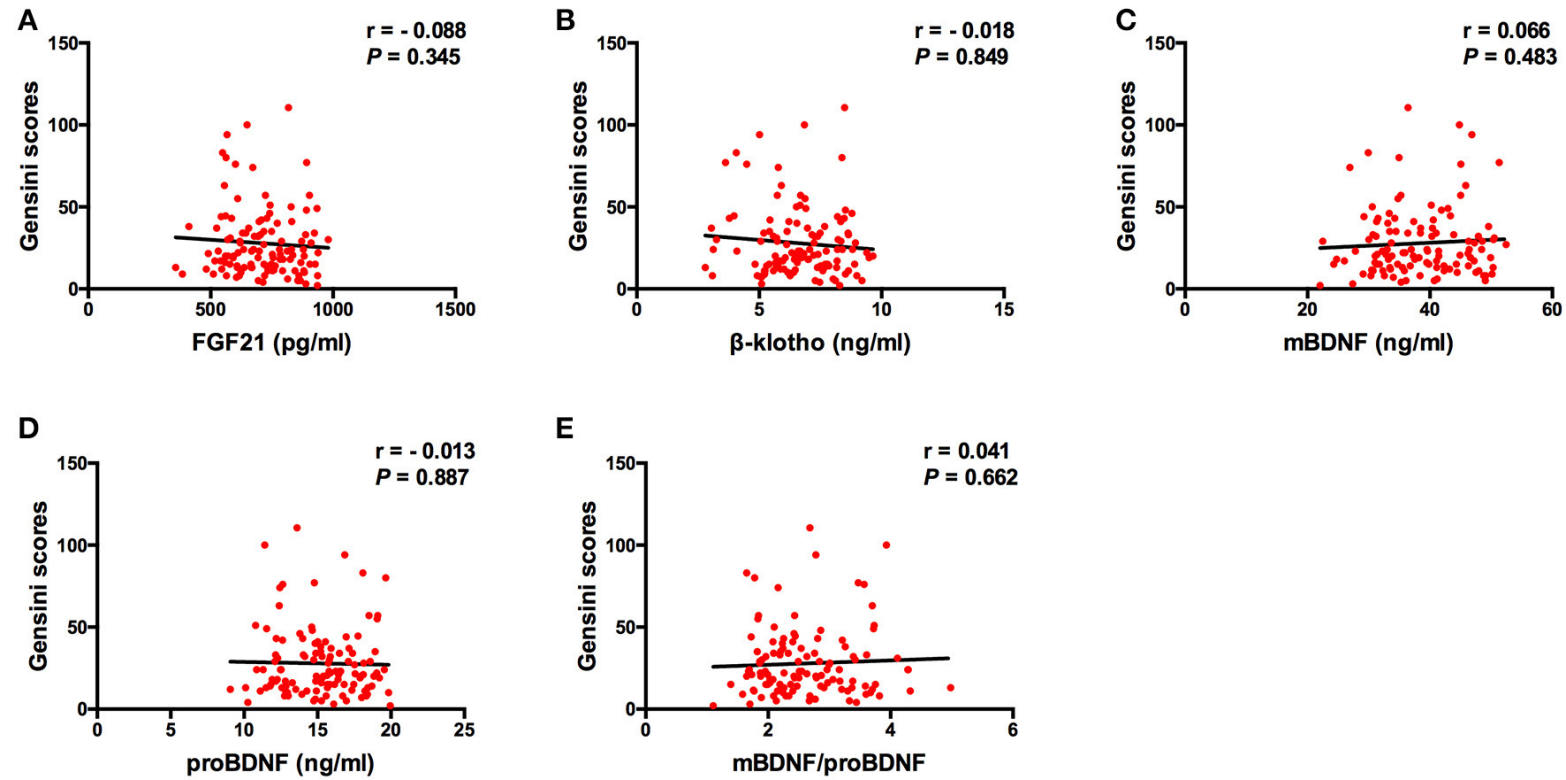
Spearman correlation analysis between serum levels of the factors and Gensini scores. BDNF, brain derived neurotrophic factor; FGF21, fibroblast growth factor 21. The correlation analysis was performed using the Spearman correlation analysis. There was no significant correlation between FGF21, β-klotho, mBDNF, proBDNF, and mBDNF/proBDNF concentrations, and the Gensini scores.

**Figure 5:**
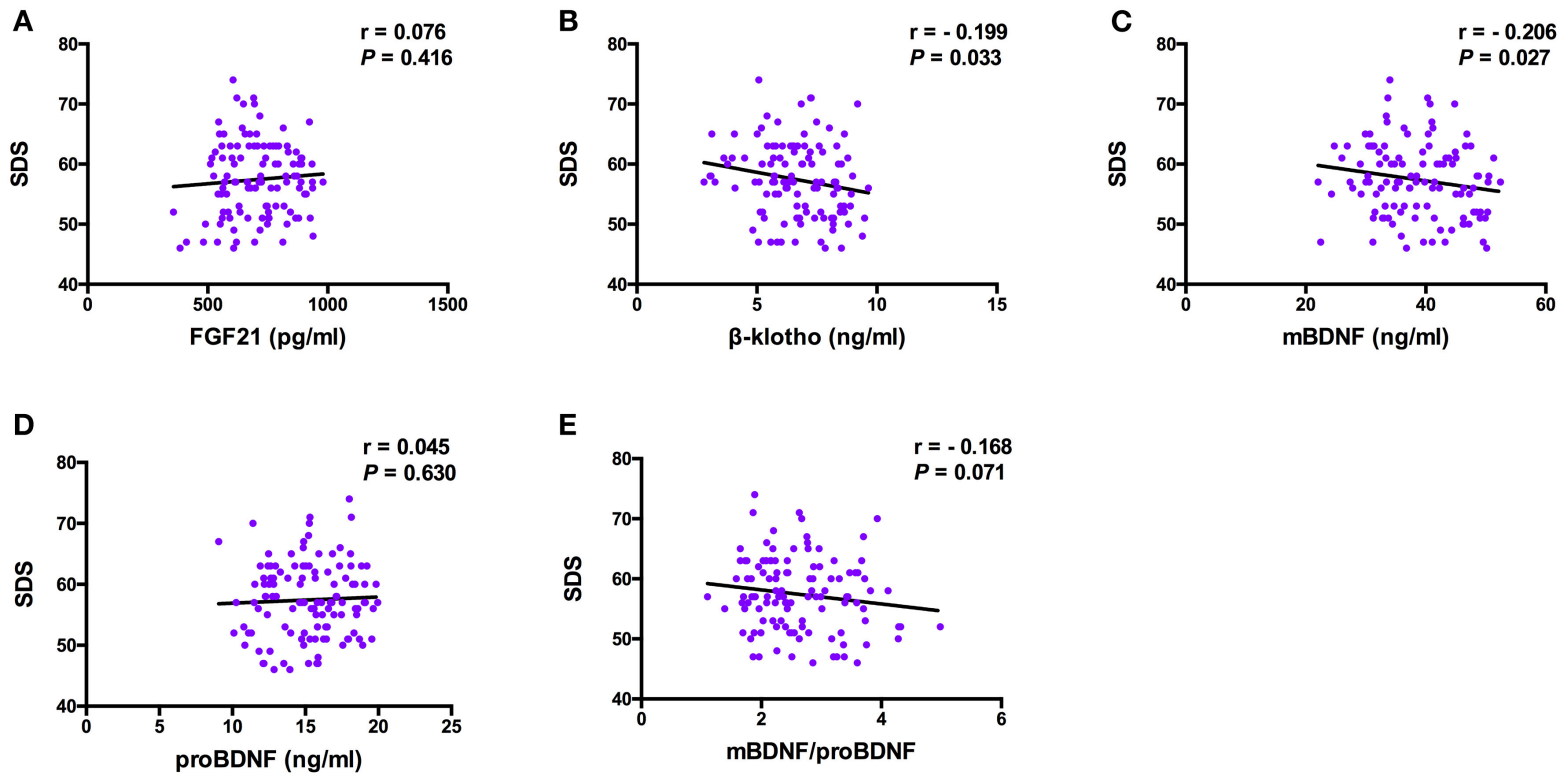
Pearson correlation analysis between serum levels of the factors and SDS. BDNF, brain derived neurotrophic factor; FGF21, fibroblast growth factor 21; SDS, self-rating depression scale. The correlation analysis was performed using the Pearson correlation analysis. β-klotho and mBDNF levels were negatively correlated with the SDS scores (*r* = −0.199, *p* = 0.033; and *r* = −0.206, *p* = 0.027, respectively).

The Spearman correlation analysis demonstrated a positive correlation between the Gensini scores and the SDS scores, and the correlation coefficient (r) was 0.168 (*p* = 0.047; Figure 3). There was no significant correlation between FGF21, β-klotho, mBDNF, proBDNF, and mBDNF/proBDNF concentrations, and the Gensini scores (Figure 4). β-klotho and mBDNF levels were negatively correlated with the SDS scores (*r* = −0.199, *p* = 0.033; and *r* = −0.206, *p* = 0.027, respectively; Figure 5). The multiple linear regression analysis showed that the serum levels of those factors were not correlated with the Gensini scores (*F* = 0.576, *p* = 0.681), and none of the variables were significant predictors of Gensini score (*p* > 0.05; Table 3). The multiple linear regression analysis built a significant model to predict SDS score based on the serum levels of those factors (*F* = 2.492, *p* = 0.047), but the coefficient of determination was low (*R*^2^ = 0.082). Significant predictor for a higher SDS score was the lower β-klotho levels serum (*B* = −0.772, *p* = 0.042; Table 4).

**Table 3:** Multiple linear regression analysis between serum levels of the factors and Gensini scores.

**Parameters**	***B***	**SE**	**95% CI**	***t***	***P*-value**
FGF21	−0.012	0.015	−0.042~0.017	−0.818	0.415
β-klotho	−1.257	1.334	−3.901~1.387	−0.942	0.348
mBDNF	0.167	0.283	−0.393~0.728	0.591	0.555
proBDNF	−0.103	0.788	−1.664~1.458	−0.131	0.896

*BDNF, brain derived neurotrophic factor; FGF21, fibroblast growth factor 21. The multiple linear regression analysis showed that there was no significant correlation between FGF21, β-klotho, mBDNF, and proBDNF concentrations, and the Gensini scores (F = 0.576, p = 0.681, R^2^ = 0.020, B0 = 40.067), and none of the variables were significant predictors of Gensini score (p > 0.05)*.

**Table 4:** Multiple linear regression analysis between serum levels of the factors and SDS.

**Parameters**	***B***	***SE***	**95% CI**	***t***	***P*-value**
FGF21	0.005	0.004	−0.003~0.014	1.240	0.217
β-klotho	−0.772	0.375	−1.515~0.029	−2.059	0.042
mBDNF	−0.140	0.079	−0.298~0.017	−1.763	0.081
proBDNF	0.031	0.013	−0.408~0.470	0.140	0.889

*BDNF, brain derived neurotrophic factor; FGF21, fibroblast growth factor 21. The multiple linear regression analysis built a significant model to predict SDS score based on the serum levels of those factors (F = 2.492, p = 0.047, B0 = 63.764), but the coefficient of determination was low (R^2^ = 0.082). Analysis of variance inflation factors (VIFs) did not demonstrate multicollinearity between factors. No violations of linearity were detected. Significant predictor for a higher SDS score was the lower β-klotho levels serum (B = −0.772, p = 0.042)*.

### Comparison of the Incidence of DS in SCAD Patients With Lower or Higher FGF21, β-klotho, mBDNF, or proBDNF Levels

According to the concentration of each of FGF21, β-klotho, mBDNF, proBDNF, and mBDNF/proBDNF, 116 patients with SCAD were further divided into a lower level group and a higher level group using a hierarchical cluster analysis (Supplementary Figures 1–4). There were 84 patients in the lower level FGF21 group and 32 patients in the higher level FGF21 group. The threshold for the higher level FGF21 group was 804.05 pg/ml. There were 48 patients in the lower level β-klotho group and 68 patients in the higher level β-klotho group, which had a threshold concentration of 6.37 ng/ml. There were 84 patients in the lower level mBDNF group and 32 patients in the higher level mBDNF group, in which the threshold concentration was 43.61 ng/ml. There were 32 cases in the lower level proBDNF group and 84 cases in the higher level proBDNF group, in which the threshold concentration was 13.15 ng/ml. Importantly, the chi-squared test demonstrated that the patients in the lower level mBDNF group had a higher incidence of DS than the higher level group (χ^2^ = 5.023, *p* = 0.025; Table 5).

**Table 5:** Incidence of depressive symptoms in SCAD patients with low or high FGF21, β-klotho, mBDNF, and proBDNF levels.

	**Without depressive symptoms (*n* = 30)**	**with depressive symptoms (*n* = 86)**	**χ^2^**	***P*-value**
**FGF21**				
Low level	23	61	0.366	0.545
High level	7	25		
**β-klotho**				
Low level	9	39	2.160	0.142
High level	21	47		
**mBDNF**				
Low level	17	67	5.023	0.025
High level	13	19		
**proBDNF**				
Low level	9	23	0.118	0.731
High level	21	63		

*BDNF, brain derived neurotrophic factor; FGF21, fibroblast growth factor 21; SCAD, stable coronary artery disease. A chi-squared test was used*.

## Discussion

The results of this study indicate a positive correlation between depression scores and the severity of coronary artery stenosis. Similarly, the serum levels of FGF21, β-klotho, mBDNF, and proBDNF are closely related to the development of DS in patients with SCAD. In addition, β-klotho and mBDNF levels were negatively correlated with the SDS scores, and the incidence of DS was significantly increased in patients with lower serum mBDNF.

It is well-recognized that the incidence of depression in patients with cardiovascular disease is significantly greater than that observed in the healthy population. Psychological disorders, such as depression, can reduce the quality of life for patients and can increase the risk of cardiovascular death (34, 35). These observations reassert that psychological intervention is necessary for patients with CHD. The Sertraline Antidepressant Heart Attack Randomized Trial found that patients with acute coronary syndrome and comorbid depression exhibited greater levels of platelet factor 4, platelet endothelial cell adhesion molecule-1, and thromboxane, which suggests enhanced platelet activation and aggregation. Platelets interact with leukocytes to stimulate cytokine release and to promote vascular intimal injury and atherosclerosis development. This process increases the incidence of CHD in healthy people, accelerates atherosclerosis, and increases mortality in patients with CHD (36, 37). Furthermore, the concentrations of interleukin-6, C-reactive protein, and tumor necrosis factor-α are increased in patients with CHD with co-morbid depression (38, 39). Therefore, the role of inflammatory processes in the physiological mechanisms of CHD with co-morbid depression has received much attention. In addition, depression-related hypothalamic-pituitary-adrenal axis hyperactivity could increase sympathetic excitability and catecholamine secretion, which leads to excessive vasoconstriction and coronary artery spasm and further aggravates myocardial ischemia (40, 41). In parallel, a decrease in the synthesis of platelet- and endothelial cell-derived nitric oxide in depressive patients limits vasodilation (42), which may exacerbate atherosclerotic plaque formation. Based on these observations, it is likely that depression and coronary atherosclerosis co-exist and interact with each other to affect the quality of life and patient prognosis.

The findings of the present study showed that patients with SCAD suffer from a variable degree of depression. The depression score is positively correlated with the severity of coronary artery stenosis. In this regard, psychological assessment should be adopted for patients with cardiac disease, especially in patients with severe coronary artery stenosis. Therefore, appropriate antidepressant drugs and active psychotherapy are necessary for symptomatic management.

Although FGF21 is a hormone-like endocrine factor that is mainly secreted by the liver, numerous studies have shown that FGF21 acts as an insulin-like growth factor to improve glucose metabolism, which would, in turn, improve serum insulin sensitivity and glucose clearance (43, 44). Additionally, FGF21 inhibits sterol regulatory element-binding protein-1 and activates uncoupling protein-1/2. In doing so, FGF21 reduces the expression of genes associated with fatty acid synthesis and fat utilization (44, 45). After virtual histology-intravascular ultrasound and FGF21 detection in 68 patients with CHD, serum FGF21 levels were significantly positively correlated with the atherosclerotic plaque burden, which can be defined as (plaque + media)/external elastic membrane (46). Unlike previous studies, we observed that patients in the SCAD group demonstrated a lower serum FGF21 compared with the HC group, indicating that FGF21 did not protect against SCAD in this study.

To exert its physiological function, FGF21 interacts with the co-receptor, β-klotho. The FGF21/β-klotho complex then binds to the plasma membrane-localized FGF receptor and activates the extracellular signal-regulated kinase 1/2 and receptor tyrosine kinases to mediate signaling cascades (47, 48). Therefore, β-klotho is the basis for the tissue-specific expression and biological function of FGF21. As an anti-aging protein, β-klotho can delay senescence through various mechanisms, including anti-oxidation, anti-senescence, and anti-autophagy. β-klotho also regulates a number of signaling pathways, including insulin-like growth factor and Wnt pathways (49). Research suggests that β-klotho plays an important role in the progression of age-related diseases such as Alzheimer's disease and neurodegeneration (50, 51). Interestingly, age is a risk factor for DS. Depression, as a heterogeneous disorder (10, 52, 53), may be associated with impaired function of various organs caused by aging (e.g., thalamic dysfunction, which leads to emotional instability) (54). In this study, serum β-klotho was significantly decreased in the group with DS. β-klotho was significantly negatively correlated with the SDS scores. To the best of our knowledge, this is the first study to demonstrate the role of β-klotho in CHD with co-morbid depression. Further detailed studies are necessary.

As a member of the neurotrophin family, BDNF is widely expressed in the adult mammalian brain in close proximity to its receptor, tropomyosin-related kinase B (TrkB). BDNF-TrkB signaling activates downstream effectors such as mitogen-activated protein kinase and phosphatidylinositol 3-kinase, thereby eliciting a protective effect on neurons (55–57). Recent studies have shown that BDNF-TrkB signaling is important in the survival of vascular endothelial cells and may promote proliferation and migration of endothelial cells in ischemic regions (28, 29, 58). In addition, exercise can promote myocardial angiogenesis in mice with myocardial infarction by activating the BDNF-TrkB axis. This pathway improves left ventricular function and has a beneficial effect on cardiac function (59, 60). Unlike mBDNF, proBDNF has a high affinity for p75NTR. ProBDNF-p75NTR activates the JNK pathway to upregulate p53 expression and initiate apoptosis. ProBDNF-p75NTR also interferes with neurotransmitter release and inhibits axonal outgrowth (61). A series of reports demonstrated that serum BDNF in depressive patients is significantly lower than that observed in the healthy population (62–64). However, few studies report the expression of mBDNF and proBDNF in patients with SCAD with co-morbid depression. Our research group previously reported that serum mBDNF was decreased in patients with SCAD, and the group with DS demonstrated significantly lower concentrations of mBDNF and mBDNF/proBDNF. Additionally, there was a negative correlation between serum mBDNF and the SDS scores. The patients in the lower mBDNF group had a higher incidence of DS than the higher level group, suggesting that a greater serum mBDNF may help to improving depression.

There are some limitations to the present study. First, DS was assessed only by using SDS. A history of depression or other psychiatric disorders was assessed by interviews, but not by professional diagnostic criteria or psychiatric examinations, which may affect the reliability of the results. Second, this study did not discuss residual confounding factors that may be relevant to the research, such as education, income, social support, and marital status. Third, owing to the short duration of hospitalization and limited maneuverability, this study was not able to determine whether psychotherapy could improve the quality of life for patients. Thus, future work is necessary to clarify these considerations. Finally, this study was a cross-sectional study with small sample size. Therefore, future population-based studies will be necessary to confirm the results.

## Conclusion

In this study, a decrease in serum FGF21 wae closely related to SCAD. Lower serum β-klotho, mBDNF, and proBDNF might indicate the development of DS in patients with SCAD and might thus represent potential diagnostic and/or therapeutic targets for patients suffering from SCAD and co-morbid depression. Hence, early recognition of abnormalities in FGF21, β-klotho, mBDNF, and proBDNF levels is crucial. Effective strategies should be formulated to improve DS and prognosis for patients.

## Data Availability Statement

The raw data supporting the conclusions of this article will be made available by the authors, without undue reservation.

## Ethics Statement

This study was approved by the Ethics Committee of the Third Affiliated Hospital of Soochow University and was registered in the Chinese Clinical Trial Registry (ChiCTR1900020594). The patients/participants provided their written informed consent to participate in this study.

## Author Contributions

LY and LZ conceived the study and participated in the design. YW, ZC, JD, and KH participated in the design, collected the data, performed statistical analyses, and drafted the manuscript. YW, ZC, and BZ conducted the analysis and developed the figures. LZ, YW, and ZC revised the manuscript. All authors read and approved the final manuscript.

## Conflict of Interest

The authors declare that the research was conducted in the absence of any commercial or financial relationships that could be construed as a potential conflict of interest.

## Supplementary Material

Supplementary Figure 1 Dendrogram of the cluster analysis of fibroblast growth factor 21.

Supplementary Figure 2 Dendrogram of the cluster analysis of β-klotho.

Supplementary Figure 3 Dendrogram of the cluster analysis of mature brain-derived neurotrophic factor.

Supplementary Figure 4 Dendrogram of the cluster analysis of brain-derived neurotrophic factor precursor.
