# Supplementary figures and images for: Serum Levels of FGF21, β-Klotho, and BDNF in Stable Coronary Artery Disease Patients With Depressive Symptoms: A Cross-Sectional Single-Center Study
Source: Front Psychiatry. 2021 Jan 21;11:587492. doi: 10.3389/fpsyt.2020.587492 (PMC7873935; doi:10.3389/fpsyt.2020.587492)

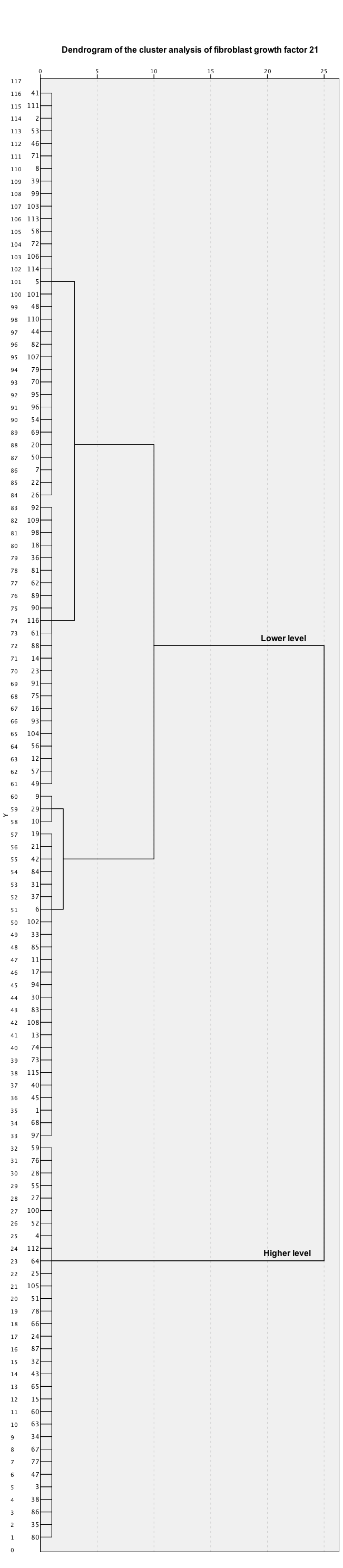

Supplement: Supplementary Figure 1 — Dendrogram of the cluster analysis of fibroblast growth factor 21. [file Image_1.TIF]

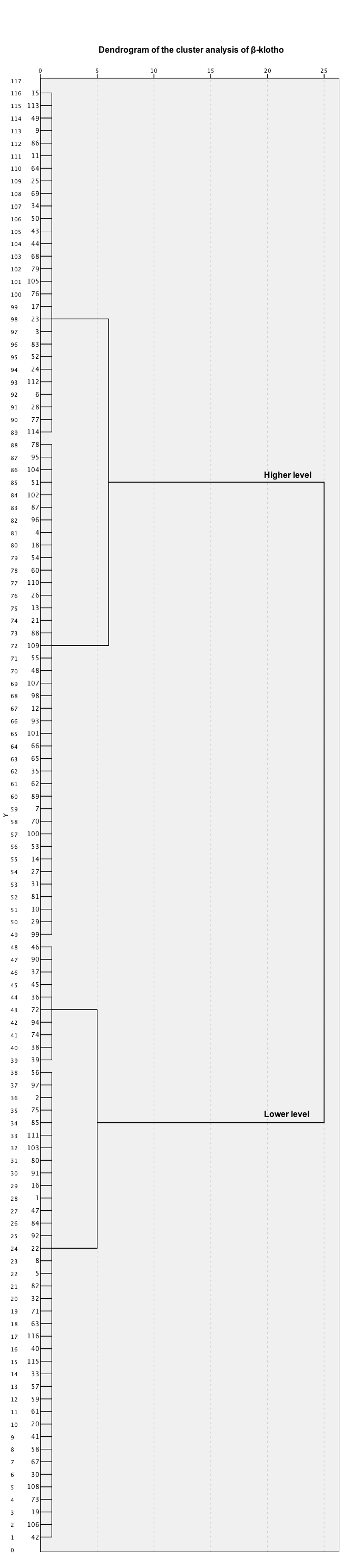

Supplement: Supplementary Figure 2 — Dendrogram of the cluster analysis of β-klotho. [file Image_2.TIF]

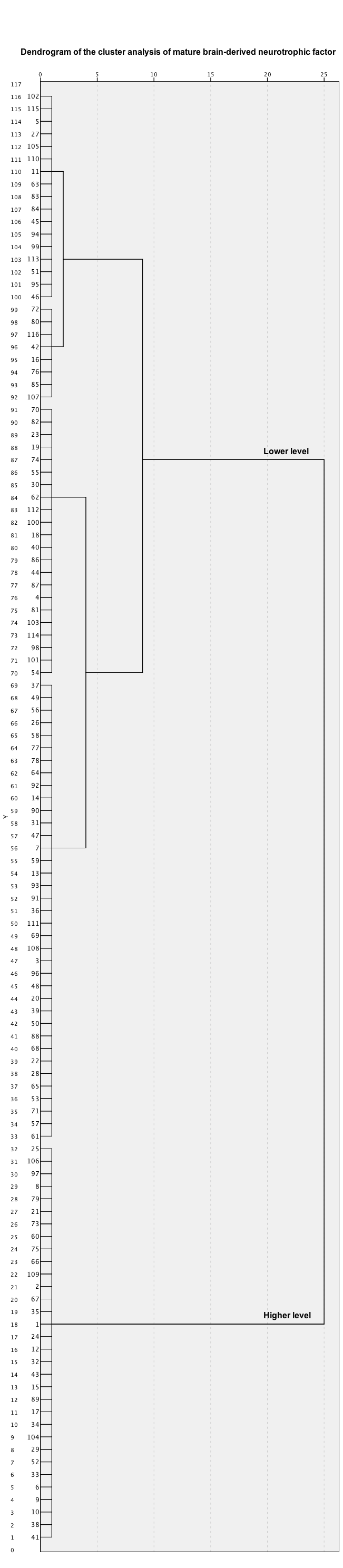

Supplement: Supplementary Figure 3 — Dendrogram of the cluster analysis of mature brain-derived neurotrophic factor. [file Image_3.TIF]

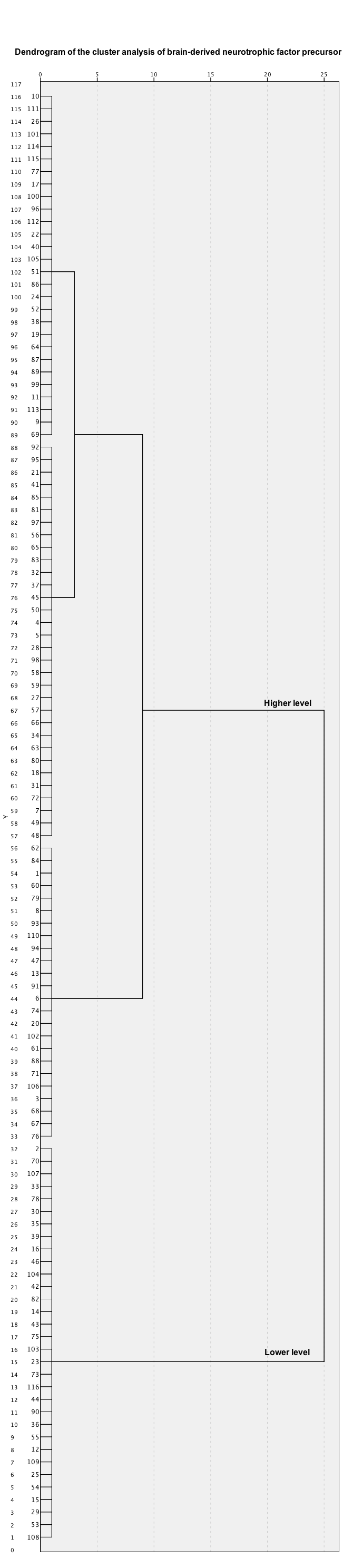

Supplement: Supplementary Figure 4 — Dendrogram of the cluster analysis of brain-derived neurotrophic factor precursor. [file Image_4.TIF]
